# Psychometric Validation of the PHQ‐9 in Ostomy Patients and Their Informal Caregivers: Evidence From A European Multicenter Study

**DOI:** 10.1002/nop2.70599

**Published:** 2026-06-16

**Authors:** Paolo Iovino, Ercole Vellone, Laura Rasero, Duilio Fiorenzo Manara, Giulia Villa

**Affiliations:** ^1^ Department of Health Sciences University of Florence Florence Italy; ^2^ Department of Biomedicine and Prevention University of Rome Tor Vergata Rome Italy; ^3^ Faculty of Nursing and Midwifery Wroclaw Medical University Wroclaw Poland; ^4^ Center for Nursing Research and Innovation Vita‐Salute San Raffaele University Milan Italy

**Keywords:** caregivers, depression, factor analysis, ostomy, patient health questionnaire 9, patient reported outcome measures, psychometrics, statistical, validation studies as topic

## Abstract

**Introduction:**

Depressive symptoms are highly prevalent among individuals with an ostomy and their informal caregivers, negatively affecting quality of life and healthcare outcomes. Although the Patient Health Questionnaire‐9 (PHQ‐9) is widely used for depression screening, its psychometric properties have not previously been examined simultaneously in ostomy patients and their informal caregivers, limiting its applicability in this clinical context.

**Aim:**

To evaluate the validity, reliability and measurement invariance of the PHQ‐9 in a European sample of ostomy patients and their informal caregivers.

**Method:**

This secondary analysis used data from a multicenter observational study including 523 ostomy patients (63.9% males, mean age 68.65 years) and 252 informal caregivers (80.6% females, mean age 58.7 years) recruited from outpatient clinics across Italy. Structural validity was examined using confirmatory factor analysis, comparing unidimensional and bidimensional models. Measurement invariance across patients and caregivers was assessed using multigroup confirmatory factor analysis. Internal consistency was assessed using omega coefficients and construct validity was tested through correlations with ostomy‐specific and generic quality of life measures.

**Results:**

Confirmatory factor analysis supported a bidimensional structure of the PHQ‐9, reflecting somatic and cognitive symptoms for both patients and caregivers, with excellent model fit. Internal consistency was adequate, with omega coefficients ranging from 0.71 to 0.88. Construct validity was supported by significant negative correlations between PHQ‐9 scores and quality of life measures. Scalar measurement invariance was established, indicating the PHQ‐9 functions equivalently in patients and caregivers.

**Conclusion:**

The PHQ‐9 is a valid and reliable instrument for assessing depressive symptoms in patients with ostomy and their caregivers and can be confidently used for depression screening in both clinical and community care settings.

**Implications for the Profession and/or Patient Care:**

Its use in routine nursing practice can support early identification and management of depression in both clinical and community care contexts.

**Impact:**

1. This is the first study validating the PHQ‐9 in patients with ostomy and their caregivers.
2. The PHQ‐9 showed excellent validity and reliability and scalar measurement invariance was demonstrated across patients and caregivers.
3. The PHQ‐9 can support nurse‐led depression screening in ostomy care settings.

**Reporting Method:**

This study was reported in accordance with the COSMIN (COnsensus‐based Standards for the selection of health Measurement INstruments) reporting guideline for studies on measurement properties.

**Patient or Public Contribution:**

No patient or public contribution.

## 1 Introduction

Ostomy is a surgical procedure included in treatments for intestinal cancer, inflammatory bowel disease and diverticular disease. According to the American Ostomy association, currently, about 1,000,000 individuals in the United States live with an ostomy, with 100,000 surgical interventions performed annually (Davis et al. 2022).

Although ostomies can increase the life expectancy of a patient, these devices often trigger significant lifestyle changes starting immediately after surgery. Various physiological, social and psychological issues challenge patients, including infection, dermatitis, difficulty travelling and dissatisfaction with appearance. Consequently, the mental health of these individuals can be affected, leading to substantial symptoms of anxiety and depression that adversely affect quality of life (Farahani et al. 2022).

Depression is among the most prevalent psychological symptoms of ostomy patients as indicated by a recent systematic review, which found that almost half of stoma patients after surgery exhibit depressive symptoms (Kovoor et al. 2023). Due to the disruptive process an ostomy placement sparks, patients are often supported by an informal caregiver throughout their psychosocial adjustment process. Caregivers are partners, family members or friends who are at the forefront of addressing the emotional and physical needs of their loved ones (Kirkland‐Kyhn et al. 2018). Those actions are identified as among the most challenging responsibilities due to the intimate nature of the task and the body image concerns often associated with stoma care (Kirkland‐Kyhn et al. 2018).

The literature indicates how informal caregivers are also at risk of depression when caring for their loved ones (Lin et al. 2024). Although ostomy‐specific prevalence data are limited, evidence from broader caregiving research highlights a substantial burden of depressive symptoms among informal caregivers. A recent umbrella review published in 2025 reported a median prevalence of depression of approximately 33% among informal caregivers of individuals with chronic conditions, with estimates ranging from 18.5% to nearly 50% across studies (Soh et al. 2025).

This may happen as a result of multifactorial causes, including the physical and emotional stress the caregiver undergoes on a daily basis and the severity of the underlying illness that the patient is affected by (e.g., cancer). Patients and informal caregivers often experience shared emotional and psychosocial challenges in the context of chronic illness management, with evidence suggesting moderate interrelatedness in psychological outcomes across these populations (Liu et al. 2023).

Despite the high prevalence of depressive symptoms among both ostomy patients and caregivers, existing research has almost exclusively assessed depression in these groups separately. To date, no study has examined the psychometric performance of a widely used depression screening tool in patients with ostomy and their caregivers, limiting the ability to conduct future dyadic assessments and comparisons. A joint consideration of patients and caregivers is particularly relevant in ostomy care, where both individuals are involved in daily disease management and may experience related psychosocial burdens (von Heymann‐Horan et al. 2018).

Promotion of psychological assessment of patients and their caregivers is therefore essential for the prevention and management of depressive symptoms. Although several valid and reliable self‐report instruments are available to screen depression, the Patient Health Questionnaire‐9 (PHQ‐9) is among the most widely used tools in both research and clinical practice due to its strong psychometric properties, brevity and ease of administration. However, to date, the PHQ‐9 has not been psychometrically validated for use in both ostomy patients and informal caregivers within the same study.

## 2 Methods

### 2.1 Aims

The objective of this study was to psychometrically test the validity and reliability of the PHQ‐9 in a sample of patients with ostomy and their caregivers.

### 2.2 Study Design and Setting

This study is a secondary psychometric analysis of data derived from a multicenter observational study conducted between 2018 and 2020. The parent study was designed to investigate self‐care behaviours and informal caregiver contribution to self‐care among individuals living with an ostomy. Data were collected in eight outpatient ostomy clinics located in northern and central Italy. All participating sites were outpatient ostomy clinics providing structured follow‐up care, with regular patient–informal caregiver contact and standardised clinical pathways for ostomy management. Other secondary analyses have already been published (Giordano et al. 2022; Iovino et al. 2023; Villa et al. 2019). This study was funded by the Centre of Excellence for Nursing Scholarship (CECRI), Rome, Italy, research Grant No. 2.16.22 and the Lega Italiana Lotta contro I Tumori (LILT), Rome, Italy, Research Grant No. 2016/U0114. The completed COSMIN checklist (Mokkink et al. 2010) is reported in Table S1 and adherence to STROBE reporting guidelines (von Elm et al. 2014) is detailed in Table S2.

### 2.3 Sampling and Participants

All participants had to be over 18 years of age, Italian‐speaking and free from severe psychiatric or cognitive impairments. All patients had to carry an ostomy at the time of data collection. No minimum time since ostomy creation was required for eligibility. Both temporary and permanent ostomies were included. All the caregivers had to be the main informal responsible individuals for the care of the patients. Specifically, an informal caregiver was defined as an unpaid family member, partner or close person who provided regular assistance and support to the patient in the management of ostomy‐related care and daily activities and who was identified by the patient as the primary caregiver. Caregivers were identified only when patients reported having a primary informal caregiver involved in ostomy care and the caregiver agreed to participate. Those who satisfied the inclusion criteria were invited to participate and those who accepted signed the informed consent form and completed a sociodemographic questionnaire and the PHQ‐9.

### 2.4 Measures

For the main study, a series of instruments were used. However, for the present analysis only the following were used.

#### 2.4.1 Patient Health Questionnaire 9

The PHQ‐9 consists of 9 items to measure the frequency of specific depressive symptoms (Kroenke et al. 2001). Responses are on a 4‐point Likert scale ranging from ‘not at all’ to ‘nearly every day’. Total scores on this scale range from 0 to 27, with higher scores indicating worse depression. Cut points of 5, 10, 15 and 20 indicate mild, moderate, moderately severe and severe depression, respectively. In this study, the PHQ‐9 was administered as a self‐report questionnaire and completed independently by both ostomy patients and their informal caregivers during routine outpatient visits. The PHQ‐9 has demonstrated sound validity and reliability properties in samples of individuals with a variety of chronic conditions (Bhana et al. 2015; Trotter et al. 2019; Udedi et al. 2019). In line with dyadic interdependence frameworks (Lyons and Lee 2018), we hypothesised that PHQ‐9 total scores would be positively correlated between patients and their caregivers, such that higher depressive symptom levels in patients would be associated with higher depressive symptom levels in caregivers.

#### 2.4.2 Stoma Specific Quality of Life

The Stoma Specific Quality of Life questionnaire (Stoma QoL) (Canova et al. 2013) consists of 20 items assessing quality of life of ostomy patients in the domains of sleep, sexual activity, relationships with family and friends and social relationships outside the family and friends. Questions are asked on a 4‐point Likert scale ranging from ‘always’ to ‘not at all’. Scores range from 20 to 80, with higher scores indicating higher QoL. Stoma QoL was used in this study to assess the convergent validity of the patient version of the PHQ‐9. Based on previous literature linking depressive symptoms with reduced quality of life in individuals living with chronic conditions (Hwang and Oh 2024; Nablawi et al. 2025), we hypothesised that higher PHQ‐9 scores would be associated with poorer ostomy‐related quality of life.

#### 2.4.3 Short Form Questionnaire 12

The SF‐12 is a 12‐item generic health‐related quality of life instrument (Ware et al. 1996), which yields two summary scores: the Physical Component Summary (PCS), which reflects physical functioning and physical health status and the Mental Component Summary (MCS), which reflects mental health, emotional well‐being and psychological functioning. Higher scores on both components indicate better perceived health status. Responses are expressed on Likert scale points ranging from ‘always’ to ‘never’. The answers allow computation of Physical Component Summary (PCS) and Mental Component Summary (MCS) scores, which range from 0 to 100 and higher scores indicate better generic QoL. The SF‐12 was used in this study to assess the convergent validity of the caregiver version of the PHQ‐9. We hypothesised that higher PHQ‐9 scores would be associated with poorer perceived health‐related quality of life (Melo et al. 2020).

### 2.5 Statistical Analysis

Analyses were conducted in four steps. First, descriptive statistics were used to summarise participant characteristics and PHQ‐9 item response distributions. Item discrimination was investigated with the item‐total correlation coefficient, with values ≥ 0.30 considered adequate. Second, structural validity was evaluated using confirmatory factor analysis (CFA) comparing a unidimensional model (Kroenke et al. 2001) with a bidimensional model distinguishing somatic and cognitive symptoms (Krause et al. 2008). Given the four‐category ordinal response format of the PHQ‐9 items, CFA models were estimated using a categorical least‐squares estimator (ULSMV), based on polychoric correlations, which is appropriate for ordered categorical indicators under the assumption of underlying continuous latent response variables (Li 2014). Model fit was evaluated using CFI and TLI (target ≥ 0.95), RMSEA (target ≤ 0.06–0.08) and SRMR (target ≤ 0.08), interpreted as guideline values rather than strict cut‐offs, particularly in categorical CFA settings (Hu and Bentler 1999). The chi‐square test was reported for completeness but not used as the primary decision criterion due to its sensitivity to sample size. For nested model comparisons under categorical estimation, the scaled chi‐square difference test was computed using the DIFFTEST procedure (i.e., mean‐ and variance‐adjusted difference testing for WLSMV/ULSMV). Δ*χ* ^2^ values reported for model comparisons reflect the DIFFTEST output (Δdf = df_restricted − df_less restricted). Factor loadings ≥ 0.30 were considered adequate (Tabachnick et al. 2013). Third, internal consistency reliability was assessed using ordinal Omega coefficient. Values < 0.70 for this index are considered supportive. Fourth, convergent validity was examined via Pearson correlations. Correlations of 0.10–0.29 were considered small, 0.30–0.49 were considered moderate and ≥ 0.50 were considered strong (Cohen et al. 2014). Finally, measurement invariance across patients and informal caregivers was evaluated via multi‐group CFA (configural, metric, scalar) following the hierarchical approach outlined by Meredith (1993), using ΔCFI ≤ 0.01 as the primary criterion for invariance (Cheung and Rensvold 2002). Multi‐group CFA treats patients and informal caregivers as two independent groups. Therefore, the effective sample sizes for invariance analyses correspond to the full available samples for each group (patients: *N* = 523; caregivers: *N* = 252). Incremental fit indices such as CFI may occasionally increase after adding equality constraints; this behaviour reflects scaling and rounding properties of the adjusted chi‐square rather than substantive improvement in model fit.

Analyses were conducted using all available data within each analytic group. Patient‐level CFA models were estimated on the full patient sample (*N* = 523) and caregiver‐level CFA models on the full caregiver sample (*N* = 252). Measurement invariance was evaluated via multi‐group CFA treating patients and caregivers as two independent groups; therefore, the effective sample sizes for invariance models corresponded to the full group‐specific samples (patients: *N* = 523; caregivers: *N* = 252). Analyses examining the association between patient and caregiver PHQ‐9 total scores were restricted to complete patient–informal caregiver pairs (*N* = 252). Prior to these tests, variables were screened for completeness and no missing data were observed in the variables included in the present analyses.

### 2.6 Sample Size

As this was a secondary analysis, no a priori sample size calculation was performed. Nevertheless, the available sample size was considered adequate for CFA and invariance testing based on commonly used rules of thumb for psychometric analyses (e.g., ≥ 10 participants per item and acceptable participant‐to‐parameter ratios) (Kline 2023), given the 9‐item structure of the PHQ‐9 and the sample sizes of patients and informal caregivers.

### 2.7 Ethical Considerations

The parent study received approval from the Institutional Review Board Committee (Ref. number 19159/18, ID2075). The principles and ethical standards outlined in the Helsinki Declaration (General Assembly of the World Medical Association 2014) were respected. Patients were informed about the study's objectives and provided written informed consent. All collected data have been securely stored and managed solely for scientific research purposes.

## 3 Results

### 3.1 Descriptive Statistics

The characteristics of the sample are presented in Table 1. Briefly, patients (*n* = 523) were mostly male (63.9%) and had an average age of 68.65 years (SD = 12.45). About three thirds were married (70%) and unoccupied or retired (67.5%). Only 17.6% lived alone and 22% reported moderate to severe depressive symptoms. Caregivers (*n* = 252), on the other hand, were predominantly female (80.6%), with an average age of 58.7 years (SD = 13.98). Most caregivers were married (80%), unoccupied or retired (58.7%) and lived with the patient (81). Overall, PHQ‐9 total scores indicated predominantly minimal to mild depressive symptoms in both patients and informal caregivers. Observed total scores ranged from [1 to 27] in patients and [1 to 24] in caregivers. Among patients, 22% reported moderate to severe depressive symptoms, while this proportion was 19.3% among caregivers. The characteristics of the items of the PHQ‐9 scale are presented in Table 2. The individual responses trended towards absence of symptoms, with significant floor effects. Item 9 had the highest floor effect both in patients and caregivers (percentage > 85). The corrected item‐total correlations were supportive, with values ≥ 0.48.

**TABLE 1:** Sociodemographic characteristics of the ostomy patients and informal caregivers included in the multicenter study in Italy.

	Patients (*n* = 523)	Caregivers (*n* = 252)
Sociodemographic characteristics		
Gender (female), *n*, %	189 (36.1)	203 (80.6)
Age (years), mean, SD	68.65 (12.45)	58.7 (13.98)
Marital status (married/partnered), *n*, %	366 (70)	200 (80)
Employment status (unoccupied/retired), *n*, %	353 (67.5)	148 (58.7)
Educational attainment (< 9 years), *n*, %	266 (50)	134 (53.2)
Perceived financial status (adequate), *n*, %	427 (81.6)	—
Living arrangements		
Live with others, *n*, %	431 (82.4)	—
Live with patient, *n*, %	—	204 (81)
Stoma characteristics		
Stoma type (colostomy), *n* %	203 (38.8)	—
Permanent stoma, *n*, %	379 (72.5)	—
Stoma‐related illness (oncologic) *n*, %	433 (82.8)	—
Stoma‐related complications during hospital stay (no), *n*, %	411 (78.6)	—
Comorbidities, *n*, %	242 (46.3)	—
Caregiver load		
Caregiving hours per week, mean, SD	—	46.46 (58.5)
Depression symptoms		
Depression severity (PHQ total score, 0–27), mean, SD	6.43 (4.76)	4.98 (5.19)
Minimal, *n*, %	230 (44)	100 (49.5)
Mild, *n*, %	178 (34)	60 (29.7)
Moderate, *n*, %	80 (15.3)	23 (11.4)
Moderately severe, *n*, %	26 (5)	14 (6.9)
Severe, *n*, %	9 (1.7)	5 (1.0)

*Note:* Total scores of this scale range from 0 to 27, with higher scores indicating greater severity of depressive symptoms. Standard cut‐off scores are commonly interpreted as follows: 0–4 = minimal, 5–9 = mild, 10–14 = moderate, 15–19 = moderately severe and ≥ 20 = severe depressive symptoms.

Abbreviations: PHQ, Patient Health Questionnaire 9; SD, standard deviation.

**TABLE 2:** Item‐level characteristics of the PHQ‐9 in ostomy patients and informal caregivers enrolled in a multicenter outpatient study in Italy.

Items' content	Patients (*n* = 523)					Caregivers (*n* = 252)				
	Not at all	Several days	More than half of the days	Nearly every day	Corrected item‐total correlation	Not at all	Several days	More than half of the days	Nearly every day	Corrected item‐total correlation
Anhedonia	295 (56.4)	147 (28.1)	54 (10.3)	27 (5.2)	0.62	135 (53.6)	81 (32.1)	27 (10.7)	9 (3.6)	0.72
2 Depressed mood	284 (54.3)	169 (32.3)	38 (7.3)	32 (6.1)	0.69	140 (55.6)	72 (28.6)	32 (12.7)	8 (3.2)	0.74
3 Sleep disturbance	229 (43.8)	166 (31.7)	58 (11.1)	70 (13.4)	0.56	107 (42.5)	84 (33.3)	32 (12.7)	29 (11.5)	0.61
4 Fatigue	147 (28.1)	227 (43.4)	85 (16.3)	64 (12.2)	0.59	85 (33.7)	100 (39.7)	40 (15.9)	27 (10.7)	0.64
5 Appetite changes	278 (53.2)	147 (28.1)	64 (12.2)	34 (6.5)	0.55	159 (63.1)	49 (19.4)	27 (10.7)	17 (6.7)	0.61
6 Low self‐esteem	410 (78.4)	76 (14.5)	22 (4.2)	15 (2.9)	0.55	207 (82.1)	25 (9.9)	13 (5.2)	7 (2.8)	0.58
7 Concentration difficulties	385 (73.6)	97 (18.5)	27 (5.2)	14 (2.7)	0.50	186 (73.8)	38 (15.1)	16 (6.3)	12 (4.8)	0.56
8 Psychomotor disturbances	417 (79.7)	75 (14.3)	17 (3.3)	14 (2.7)	0.48	207 (82.1)	25 (9.9)	17 (6.7)	3 (1.2)	0.69
9 Suicidal ideation	446 (85.3)	50 (9.6)	14 (2.7)	13 (2.5)	0.51	230 (91.3)	11 (4.4)	6 (2.4)	5 (2)	0.49

*Note:* Data are absolute numbers and percentages.

### 3.2 Factorial Structure

#### 3.2.1 Patient Model

The initial CFA (Model 1) yielded marginal fit indices: *χ* ^2^ (27, *N* = 523) = 116.82, *p* < 0.001; RMSEA = 0.080 (90% CI = [0.065, 0.095]; *p*(RMSEA < 0.05) = 0.001); CFI = 0.96; TLI = 0.94; SRMR = 0.052. This model was compared with a two‐factor CFA (Model 2), resulting in better‐fit indices: *χ* ^2^ (26, *N* = 523) = 106.67, *p* < 0.001; RMSEA = 0.077 (90% CI = [0.062, 0.093]; p(RMSEA < 0.05) = 0.002); CFI = 0.96; TLI = 0.95; SRMR = 0.049. This model yielded a statistically significant scaled chi‐square difference test when compared with the two‐factor model: Satorra Bentler *χ* ^2^ (1) = 10.31, *p* = 0.001, indicating the bidimensional solution as the best model (Table S3). Factor loadings were all significant and high (Table 3).

**TABLE 3:** Standardised factor loadings of the bidimensional PHQ‐9 model in patients and informal caregivers.

Item	Patient version (*n* = 523)	Caregiver version (*n* = 252)
Factor 1. Somatic symptoms		
(3) Sleep disturbance	0.70 (0.03)	0.78 (0.04)
(4) Fatigue	0.75 (0.03)	0.81 (0.04)
(5) Appetite changes	0.71 (0.03)	0.77 (0.05)
Factor 2. Cognitive symptoms		
(1) Anhedonia	0.76 (0.03)	0.87 (0.03)
(2) Depressed mood	0.85 (0.02)	0.83 (0.03)
(6) Low self‐esteem	0.75 (0.04)	0.80 (0.04)
(7) Concentration difficulties	0.71 (0.04)	0.73 (0.05)
(8) Psychomotor disturbances	0.67 (0.04)	0.90 (0.04)
(9) Suicidal ideation	0.76 (0.04)	0.80 (0.07)

*Note:* Standard errors are in brackets.

#### 3.2.2 Caregiver Model

The initial CFA (Model 1) yielded acceptable fit indices: *χ* ^2^ (27, *N* = 253) = 65.51, *p* < 0.001; RMSEA = 0.075 (90% CI = [0.052, 0.099]; *p*(RMSEA < 0.05) = 0.037); CFI = 0.98; TLI = 0.97; SRMR = 0.054. This model was compared with a two‐factor CFA (Model 2), resulting in better fit indices: *χ* ^2^ (26, *N* = 253) = 56.12, *p* < 0.001; RMSEA = 0.068 (90% CI = [0.043, 0.092]; *p*(RMSEA < 0.05) = 0.108); CFI = 0.98; TLI = 0.97; SRMR = 0.048. This model yielded a statistically significant scaled chi‐square difference test when compared with the two‐factor model: Satorra Bentler *χ* ^2^(1) = 8.70, *p* = 0.003, indicating the bidimensional solution as the best model (Table S3). Factor loadings were all significant and high (Table 3).

### 3.3 Reliability and Construct Validity

For the patient version of the PHQ‐9, omega coefficients for the somatic and cognitive factors were adequate at 0.71 and 0.80, respectively. This coefficient was also adequate when computed for the whole scale (0.85). For the caregiver version of the PHQ‐9, omega coefficients were 0.77 for the somatic factor and 0.86 for the cognitive factor. When computed for the whole scale, this index was also adequate at 0.88.

When the patient PHQ‐9 scores were compared with the stoma QOL scores, we found that those with higher depressive symptoms were more likely to have poorer QOL, compared to the others (ρ = −0.20, *p* < 0.001). Similarly, when the caregiver PHQ‐9 scores were correlated with the scores of the PCS and MCS of the SF‐12 we found that those with higher depressive symptoms tended to report poorer physical and mental QOL compared to their counterparts (MCS: *ρ* = −0.16, *p* = 0.012; PCS: *ρ* = −0.41, *p* < 0.001). Finally, PHQ‐9 total scores were positively correlated among matched patient–caregiver pairs (*r* = 0.34, *p* < 0.001), indicating that higher levels of depressive symptoms in patients were associated with higher depressive symptoms in their caregivers.

### 3.4 Measurement Invariance

The results of the measurement invariance testing are reported in Table 4. The baseline configural model showed acceptable fit (CFI = 0.973), supporting a comparable factorial structure across patients and informal caregivers. Constraining factor loadings to equality across groups (metric invariance) did not change model fit (CFI = 0.973, ΔCFI = 0.000), supporting metric invariance. When equality constraints were additionally imposed on item intercepts (scalar invariance), model fit further improved (CFI = 0.989, ΔCFI = +0.016), supporting scalar invariance across groups.

**TABLE 4:** Measurement invariance testing across patients (*n* = 523) and caregivers (*n* = 252) according to Meredith's framework.

Model	*χ* ^2^ (df)	Δ*χ* ^2^ (Δdf)	CFI	ΔCFI	RMSEA	SRMR	Description
Configural	141.142 (52)	—	0.973	—	0.067	0.049	Baseline model: same factor structure across groups
Metric	149.928 (61)	8.786 (9)	0.973	0	0.061	0.060	Factor loadings constrained equal across groups
Scalar	124.183 (88)	25.745 (27)	0.989	0.016	0.033	0.062	Factor loadings and intercepts constrained equal

Abbreviations: ΔCFI, Change in Comparative Fit Index; Δdf, chi‐square difference; *χ* ^2^, chi‐square statistic; CFI, Comparative Fit Index; df, degrees of freedom; RMSEA, Root Mean Square Error of Approximation; SRMR, Standardised Root Mean Square Residual.

## 4 Discussion

The aim of this study was to test the psychometric properties of the PHQ‐9 in a sample of patients with ostomy and their caregivers, with particular attention to its factorial structure, reliability, construct validity, measurement invariance and within‐group associations. Overall, the findings support the use of the PHQ‐9 in this population. Structural validity analyses indicated that a bidimensional model distinguishing somatic and cognitive symptoms provided a better fit than a unidimensional solution in both patients and informal caregivers. The PHQ‐9 also showed satisfactory internal consistency and construct validity, as evidenced by coherent associations with quality‐of‐life measures. Measurement invariance analyses further demonstrated that the instrument functions equivalently across patients and caregivers, allowing meaningful comparisons between patients and informal caregivers at the group level. Finally, the positive correlation between patient and caregiver PHQ‐9 scores supports the hypothesised interconnected psychological experiences in these populations.

This is the first study that tested this instrument in the context of ostomy care. Considering the vulnerability of the patients with ostomy and their caregivers in experiencing depression and that this outcome is a detrimental prognostic factor for their QoL, we provided evidence that the PHQ‐9 can be used confidently to promote initiatives of mental health screening in inpatient, outpatient and community settings.

The study sample appears to reflect a relatively homogeneous outpatient population characterised by generally low levels of depressive symptoms in both patients and informal caregivers. This pattern is consistent with the clinical setting of the participating centres, which provide routine outpatient follow‐up care rather than acute or psychiatric services. These characteristics likely contributed to the predominance of low symptom severity, as further supported by the observed floor effects at the item level. While limited symptom severity may constrain variability in depressive symptom expression, it also reflects a realistic clinical context in which the PHQ‐9 is commonly used for screening purposes. From a clinical perspective, the PHQ‐9 allows a straightforward interpretation of depressive symptom severity based on established cut‐off scores. In this study, the distribution of participants across severity categories and the proportion of individuals scoring ≥ 10—commonly considered indicative of clinically relevant depressive symptoms—provide useful information for screening purposes in ostomy care settings. For nurses involved in outpatient follow‐up, these results support the use of the PHQ‐9 as a brief screening tool to identify patients and informal caregivers who may benefit from further psychological assessment or referral.

In line with the generally low level of depressive symptoms observed in this ambulatory sample, floor effects were evident for several PHQ‐9 items, particularly those reflecting more severe or less frequently endorsed symptoms. We ruled out measurement issues with the PHQ‐9 as a cause for this pattern, since all response categories were utilised across the sample, indicating that the instrument's full range was accessible and functioning properly. These floor effects are therefore more likely attributable to the characteristics of our sample, which consisted predominantly of individuals with low symptom severity.

We found that both the patient and caregiver versions of the PHQ‐9 had a bidimensional factorial structure. This contrasts with the unidimensional structure originally proposed by the scale's developers (Kroenke et al. 2001). Nonetheless, our findings are consistent with other studies that have identified a two‐factor model on the PhQ‐9, typically separating somatic and cognitive‐affective symptom clusters. Several factors may explain these discrepancies.

First, our sample reflects a homogenous population with low depression. It is possible that the ostomy context and the underlying disease have sparked somatic symptoms, which ultimately reflected a separate somatic factor. Second, both the characteristics of the homogeneous population and low depressive symptoms may have led to a restriction in variance of the items; thus, reduction of the covariances among the indicators (Fabrigar et al. 1999). When the PHQ‐9 is administered in mixed populations, it is more likely that a one‐factor solution emerges, as a result of the greater items' covariation (Petersen et al. 2015). Thus, sample characteristics, including clinical status and symptoms, appear to play a critical role in the factorial validity of the PHQ‐9.

Although the factorial analyses supported a bidimensional structure distinguishing somatic and cognitive symptoms, these dimensions should not be interpreted as independent clinical subscales. The PHQ‐9 was originally developed and validated as a unidimensional screening instrument and the somatic and cognitive components identified in factor analytic models primarily reflect latent structure rather than separate clinical constructs intended for standalone interpretation.

The construct validity of the PHQ‐9 was established through the findings of significant positive correlations between its scores and both specific and general QOL measures. This aligns with existing research and highlights the negative impact that depressive symptoms can have on different facets of an individual's health and overall well‐being when the dyad is involved in ostomy management (Lin et al. 2024; Rafiei et al. 2020; Shrestha et al. 2022).

Our findings also indicate an excellent internal consistency, which is consistent with the results of psychometric analyses conducted in other samples (Hinz et al. 2016; Tibubos et al. 2021). This reinforces the strength that the total scores obtained from the PHQ‐9 are likely to be reliable in this population. Extraction of partial scores from the cognitive and somatic factors is also recommended in case clinicians need a more nuanced description of the depressive symptoms in patients and their caregivers.

Notably, we also found invariance of the PHQ‐9 scale across ostomy patients and their caregivers. Achieving full measurement invariance of the PHQ‐9 across these groups provides strong psychometric support for comparing depressive symptom scores between these individuals. Scalar invariance, in particular, ensures that both the factor structure and the scale metric are equivalent, meaning that any observed differences in total or latent scores reflect true differences in underlying depressive symptoms rather than measurement bias. This finding is crucial in the context of dyadic research, where both patients and caregivers are conceptualised as an interdependent unit rather than isolated individuals (Lyons and Lee 2018). Dyadic frameworks such as the Actor‐Partner Interdependence Model (APIM) (Fitzpatrick et al. 2016) often rely in comparable measures across members of the dyad, to assess how one member's psychological state affects the other. By establishing such invariance, we have provided a basis for future studies applying formal dyadic analytical approaches (e.g., APIM) to investigate interdependence mechanisms between patients and caregivers.

### 4.1 Limitations and Strengths

This study has a few limitations. First, there are potential generalizability issues, because although the patients and caregivers were recruited in several outpatient clinics across Italy, our sample had mild depression. Future research should include more clinically heterogeneous samples, such as inpatients or individuals receiving specialised psychological care, to examine the performance of the PHQ‐9 across a broader severity spectrum. Second, the cross‐sectional design precludes assessment of responsiveness and sensitivity to change; longitudinal studies are therefore needed to evaluate the stability and responsiveness of the PHQ‐9 over time in ostomy patient–caregiver dyads. Third, although the study included paired patient–caregiver data, the analytical approach did not formally account for interdependence between dyad members. The primary psychometric analyses were conducted at the individual level and the association between patients and caregivers was examined using simple correlational methods. Future research should apply formal dyadic modelling approaches (e.g., APIM or multilevel SEM) to more rigorously investigate interdependence mechanisms. Fourth, replication in different cultural and healthcare contexts would help to confirm the generalizability of the present findings. Finally, criterion validity could not be assessed because a diagnostic gold standard for depression (e.g., a structured clinical interview) was not available in the parent study. Future studies should therefore examine the diagnostic accuracy of the PHQ‐9 against clinical diagnostic interviews to establish optimal cut‐off scores and sensitivity–specificity parameters in this population.

This study also has several methodological strengths that enhance the validity and credibility of the findings. First, a comprehensive psychometric validation process was applied, encompassing the assessment of structural validity, internal consistency, construct validity and measurement invariance, in line with established recommendations for measurement studies. Second, the inclusion of both patients and caregivers within the same study framework allowed us to examine the psychometric performance of the PHQ‐9 across these related populations and to explore associations between their depressive symptom levels. Third, the multicenter outpatient design increases the generalizability of the findings to routine ostomy care settings. Finally, the transparent reporting of item‐level distributions and model fit indices strengthens the robustness and interpretability of the results.

### 4.2 Implications for Nursing Practice

The findings of this study have direct relevance for nursing professionals engaged in ostomy care across inpatient, outpatient and community settings. Given the prevalence of depressive symptoms among both patients and their caregivers, routine screening for mental health conditions is essential. The PHQ‐9, validated here for the first time in this population, offers a brief, cost‐effective and psychometrically robust solution that can be easily integrated into nursing assessments. Its bidimensional structure allows nurses not only to detect overall depressive symptomatology but also to distinguish between cognitive and somatic components of depression, thus enabling more targeted psychosocial interventions. Furthermore, the demonstrated measurement invariance supports its use in dyadic care models, facilitating comparative monitoring and dyadic interventions tailored to both members. Nurse‐led initiatives for early detection and referral can significantly enhance patient outcomes, caregiver well‐being and care continuity.

## 5 Conclusion

This study provides evidence supporting the psychometric validity of the PHQ‐9 for assessing depressive symptoms across both patients and informal caregivers within outpatient care settings. The findings confirm a bidimensional factorial structure distinguishing somatic and cognitive symptoms, satisfactory reliability and coherent construct validity, together with measurement invariance across patients and caregivers, enabling future meaningful comparisons within these dyads. The generally low level of depressive symptoms observed in this ambulatory and relatively homogeneous population, along with the presence of floor effects, reflects the clinical context in which the instrument is most commonly used for screening purposes and may have influenced the observed factor structure. Importantly, the positive association between patient and caregiver depressive symptoms highlights the relevance of adopting a dyadic perspective in ostomy care. Overall, these results support the use of the PHQ‐9 as a reliable and valid screening tool for depressive symptoms in routine ostomy follow‐up, while underscoring the importance of considering population characteristics and care settings when interpreting psychometric findings.

## Author Contributions

Conceptualization: Ercole Vellone, Giulia Villa and Paolo Iovino. Methodology: Ercole Vellone and Paolo Iovino. Formal analysis: Paolo Iovino. Investigation: Giulia Villa. Resources: Giulia Villa. Data curation: Ercole Vellone and Giulia Villa. Writing – original draft preparation: Paolo Iovino. Writing – review and editing: Ercole Vellone, Giulia Villa, Paolo Iovino. Visualisation: Paolo Iovino. Supervision: Duilio Fiorenzo Manara and Laura Rasero. All authors have read and agreed to the published version of the manuscript. Please turn to the CRediT taxonomy for the term explanation. Authorship must be limited to those who have contributed substantially to the work reported.

## Funding

This work was supported by the Centre of Excellence for Nursing Scholarship (2.16.22) and Lega Italiana Lotta contro I Tumori (2016/U0114).

## Ethics Statement

This research received approval from the Institutional Review Board Committee (Ref. number 19159/18, ID2075). The principles and ethical standards outlined in the Helsinki Declaration were respected. Patients were informed about the study's objectives and provided written informed consent. All collected data have been securely stored and managed solely for scientific research purposes.

## Conflicts of Interest

The authors declare no conflicts of interest.

## Supporting information


**Table S1:** COSMIN checklist for reporting studies on measurement properties.
**Table S2:** STROBE checklist for cross‐sectional studies.
**Table S3:** Comparison of unidimensional and bidimensional PHQ‐9 models in patients and informal caregivers.

## Data Availability

The data that support the findings of this study are available on request from the corresponding author. The data are not publicly available due to privacy or ethical restrictions.
