# Supplementary material for: Psychometric Validation of the PHQ‐9 in Ostomy Patients and Their Informal Caregivers: Evidence From A European Multicenter Study
Source: Nurs Open. 2026 Jun 16;13(6):e70599. doi: 10.1002/nop2.70599 (PMC13271098; doi:10.1002/nop2.70599)
Supplement: Supplementary file 1 — Table S1: COSMIN checklist for reporting studies on measurement properties. Table S2: STROBE checklist for cross‐sectional studies. Table S3: Comparison of unidimensional and bidimensional PHQ‐9 models in patients and informal caregivers. [file NOP2-13-e70599-s001.docx]

**Supplementary Table S1.** COSMIN checklist for reporting studies on measurement properties.

| COSMIN domain | COSMIN item | Reported | Section in manuscript | Notes |
| --- | --- | --- | --- | --- |
| PROM development | Construct definition | N.A. | – | PHQ-9 is an established instrument and was not newly developed in this study |
|  | Target population described | Yes | Methods – Sampling and participants | Ostomy patients and informal caregivers |
|  | Context of use described | Yes | Introduction; Methods – Study design and setting | Outpatient ostomy clinics |
| Content validity | Relevance of items | N.A. | – | Content validity previously established in original PHQ-9 validation |
|  | Comprehensiveness | N.A. | – | Instrument not newly developed |
|  | Comprehensibility | N.A. | – | Instrument already validated and widely used |
| Structural validity | Factorial structure tested | Yes | Methods – Statistical analysis; Results – Factorial structure | CFA comparing unidimensional vs bidimensional models |
|  | Appropriate estimator used | Yes | Methods – Statistical analysis | ULSMV estimator for ordinal items |
|  | Model fit indices reported | Yes | Results – Factorial structure; Supplementary Table S1 | CFI, TLI, RMSEA, SRMR |
|  | Factor loadings reported | Yes | Results – Table 3 | All standardized loadings ≥0.30 |
| Internal consistency | Reliability coefficient reported | Yes | Results – Reliability and construct validity | Ordinal omega coefficients |
|  | Reliability criteria defined | Yes | Methods – Statistical analysis | Threshold ≥0.70 |
|  | Reliability per dimension | Yes | Results – Reliability and construct validity | Somatic and cognitive dimensions |
| Measurement invariance / cross-group validity | Groups defined a priori | Yes | Methods – Statistical analysis | Patients vs informal caregivers |
|  | Configural invariance tested | Yes | Results – Measurement invariance | MG-CFA |
|  | Metric invariance tested | Yes | Results – Measurement invariance | ΔCFI criterion |
|  | Scalar invariance tested | Yes | Results – Measurement invariance | Scalar invariance established |
| Construct validity (hypothesis testing) | A priori hypotheses specified | Yes | Methods – Statistical analysis | Depression expected to correlate with QoL |
|  | Direction of associations defined | Yes | Methods – Statistical analysis | Higher depression associated with poorer QoL |
|  | Appropriate statistical tests | Yes | Methods – Statistical analysis | Pearson correlations |
|  | Hypotheses confirmed | Yes | Results – Reliability and construct validity | Associations with Stoma QoL and SF-12 |
| Criterion validity | Gold standard available | N.A. | – | No diagnostic interview used |
|  | Comparison with gold standard | N.A. | – | Not applicable |
| Responsiveness | Longitudinal change evaluated | No | – | Cross-sectional design |
| Interpretability | Score distribution reported | Yes | Results – Descriptive statistics; Table 1 | Depression severity categories |
|  | Floor/ceiling effects described | Yes | Results – Descriptive statistics | Floor effects observed |
|  | Clinical meaning discussed | Yes | Discussion | Implications for screening |
| Generalizability | Sample characteristics reported | Yes | Results – Descriptive statistics; Table 1 | Sociodemographic and clinical variables |
|  | Setting described | Yes | Methods – Study design and setting | Multicenter outpatient clinics |
| Statistical methods | Missing data handling | Yes | Methods – Statistical analysis | Dataset screened for completeness |
|  | Software / analysis approach described | Yes | Methods – Statistical analysis | CFA and MG-CFA procedures described |
| Reporting quality | Results reported per analysis | Yes | Results section | All planned analyses reported |
|  | Limitations discussed | Yes | Discussion – Limitations and strengths | Generalizability and cross-sectional design |

**Note.** COSMIN = Consensus-based Standards for the selection of health Measurement INstruments. The table indicates whether each COSMIN reporting item was addressed in the manuscript and specifies the corresponding section where the information is reported.

**Supplementary Table S2.** STROBE checklist for cross-sectional studies

| STROBE item | Recommendation | Section in manuscript |
| --- | --- | --- |
| Title and abstract |  |  |
| 1a | Indicate the study design with a commonly used term in the title or abstract | Abstract – Methods |
| 1b | Provide an informative and balanced summary of what was done and what was found | Abstract |
| Introduction |  |  |
| 2 | Explain the scientific background and rationale for the investigation | Introduction |
| 3 | State specific objectives and hypotheses | Methods – Aims |
| Methods |  |  |
| 4 | Present key elements of study design early in the paper | Methods – Study design and setting |
| 5 | Describe the setting, locations, and relevant dates including recruitment period | Methods – Study design and setting |
| 6a | Give eligibility criteria and sources/methods of participant selection | Methods – Sampling and participants |
| 6b | For matched studies, give matching criteria | Not applicable |
| 7 | Clearly define outcomes, exposures, predictors, and confounders | Methods – Measures |
| 8 | For each variable of interest, give data sources and measurement methods | Methods – Measures |
| 9 | Describe efforts to address potential sources of bias | Methods – Sampling and participants; Discussion – Limitations |
| 10 | Explain how the study size was determined | Methods – Sample size |
| 11 | Explain how quantitative variables were handled in the analyses | Methods – Statistical analysis |
| 12a | Describe all statistical methods used | Methods – Statistical analysis |
| 12b | Describe methods used to examine subgroups and interactions | Methods – Statistical analysis (measurement invariance analyses) |
| 12c | Explain how missing data were addressed | Methods – Statistical analysis |
| 12d | Describe analytical methods taking account of sampling strategy | Methods – Sampling and participants |
| 12e | Describe sensitivity analyses | Not applicable |
| Results |  |  |
| 13a | Report numbers of individuals at each stage of the study | Results – Descriptive statistics |
| 13b | Give reasons for non-participation | Not available |
| 13c | Consider use of a flow diagram | Not applicable |
| 14a | Give characteristics of study participants | Results – Table 1 |
| 14b | Indicate number of participants with missing data | Results – Descriptive statistics |
| 15 | Report outcome events or summary measures | Results – Descriptive statistics |
| 16a | Provide estimates and precision measures | Results – Tables 3 and 4 |
| 16b | Report category boundaries when continuous variables categorized | Results – Table 1 (PHQ-9 severity categories) |
| 16c | If relevant, translate estimates into absolute risk | Not applicable |
| 17 | Report other analyses done (e.g., subgroup analyses) | Results – Measurement invariance |
| Discussion |  |  |
| 18 | Summarize key results with reference to study objectives | Discussion – First paragraph |
| 19 | Discuss limitations of the study | Discussion – Limitations and strengths |
| 20 | Provide interpretation considering objectives and limitations | Discussion |
| 21 | Discuss generalizability of findings | Discussion – Limitations and strengths |
| Other information |  |  |
| 22 | Give the source of funding and role of funders | Methods – Study design and setting |

**Note.** STROBE = Strengthening the Reporting of Observational Studies in Epidemiology. This checklist summarizes how the present cross-sectional psychometric study adheres to STROBE reporting recommendations, with links to the corresponding manuscript sections.

**Supplementary Table S3.** Comparison of unidimensional and bidimensional PHQ-9 models in patients and informal caregivers

| Group | Model | χ² (df) | CFI | TLI | RMSEA (90% CI) | SRMR | Δχ² (Δdf) | ΔCFI |
| --- | --- | --- | --- | --- | --- | --- | --- | --- |
| Patients | 1-factor | 116.82 (27) | 0.96 | 0.94 | 0.080 | 0.052 | – | – |
| Patients | 2-factor | 106.67 (26) | 0.96 | 0.95 | 0.077 | 0.049 | 10.31 (1)* | 0.00 |
| Caregivers | 1-factor | 65.51 (27) | 0.98 | 0.97 | 0.075 | 0.054 | – | – |
| Caregivers | 2-factor | 56.12 (26) | 0.98 | 0.97 | 0.068 | 0.048 | 8.70 (1)* | 0.00 |

**Note.** CFI = Comparative Fit Index; TLI = Tucker–Lewis Index; RMSEA = Root Mean Square Error of Approximation; SRMR = Standardized Root Mean Square Residual. Lower RMSEA and SRMR values indicate better model fit.

**Legend.** * Satorra–Bentler scaled χ² difference test.
